# Preliminary qualification of a novel, hypoxic-based radiologic signature for trans-arterial chemoembolization in hepatocellular carcinoma

**DOI:** 10.1186/s12885-018-4120-4

**Published:** 2018-02-20

**Authors:** David J. Pinato, Madhava Pai, Isabella Reccia, Markand Patel, Alexandros Giakoustidis, Georgios Karamanakos, Azelea Rushd, Shiraz Jamshaid, Alberto Oldani, Glenda Grossi, Mario Pirisi, Paul Tait, Rohini Sharma

**Affiliations:** 1 Department of Surgery and Cancer, Imperial College London, Hammersmith Hospital, Du Cane Road, London, W120HS UK; 2 Department of Hepatobiliary Surgery, Imperial College NHS Trust, Hammersmith Hospital, Du Cane Road, London, W120HS UK; 3 Division of Experimental Medicine, Imperial College London, Hammersmith Hospital, Du Cane Road, London, W120HS UK; 4 Department of Radiology, Imperial College NHS Trust, Hammersmith Hospital, Du Cane Road, London, W120HS UK; 5 Department of Health Sciences, Università degli Studi del Piemonte Orientale “A.Avogadro”, via Solaroli 17, 28100 Novara, Italy; 6 Department of Translational Medicine, Università degli Studi del Piemonte Orientale “A. Avogadro”, Via Solaroli 17, 28100 Novara, Italy; 7 Interdisciplinary Research Center of Autoimmune Diseases, Università degli Studi del Piemonte Orientale “A. Avogadro”, Via Solaroli 17, 28100 Novara, Italy

**Keywords:** Prognosis, Hepatocellular carcinoma, Transarterial chemoembolisation, Prognostic index, Survival

## Abstract

**Background:**

Survival advantage following trans-arterial chemoembolization (TACE) is variable in patients with hepatocellular carcinoma (HCC). We combined pre-TACE radiologic features to derive a novel prognostic signature in HCC.

**Methods:**

A multi-institutional dataset of 98 patients was generated from two retrospective cohorts from United Kingdom (65%) and Italy (36%). The prognostic impact of a number baseline imaging parameters was assessed and factors significant on univariate analysis were combined to create a novel radiologic signature on multivariable analyses predictive of overall survival (OS) following TACE.

**Results:**

Median OS was 15.4 months. Tumour size > 7 cm (*p* < 0.001), intra-tumour necrosis (ITN) (*p* = 0.02) and arterial ectatic neovascularisation (AEN) (*p* = 0.03) emerged as individual prognostic factors together with radiologic response (*p* < 0.001) and elevated alpha-fetoprotein (AFP) (*p* = 0.01). Combination of tumour size > 7 cm, ITN and AEN identified patients with poor prognosis (*p* < 0.001).

**Conclusions:**

We identified a coherent signature based on commonly available imaging biomarkers likely to be reflective of differential patterns of relative hypoxia and neovascularisation. Large tumours displaying AEN and ITN are characterised by a shorter survival after TACE.

## Background

Trans-arterial chemo-embolisation (TACE) is universally recognised as a suitable therapy to improve the survival of patients with hepatocellular carcinoma (HCC) who cluster into the “intermediate” Barcelona Clinic Liver Cancer (BCLC) stage [1].

Heterogeneity in survival is nonetheless wide and originates from numerous patient as well as treatment-related factors [2]. In clinical practice TACE is often performed sequentially until technically feasible and/or extra-hepatic or portal vein invasion (PVI) develops [3]. The lack of shared criteria to define chemoembolization failure, however, makes it difficult for clinicians to estimate long-term benefits from repeated loco-regional treatments, a point of major concern due to the significant rate of morbidity and mortality attributable to TACE in a palliative population [4].

It is felt that TACE might be futile in a proportion of patients displaying adverse prognostic features, a concept that has led to the qualification of novel prognostic algorithms in this population [5], none of which has however entered the clinic due to concerns over external validity [6].

Quantification of tumour burden and PVI are central elements of most HCC staging systems [7]. Additional imaging features of HCC reflecting vascularity and growth pattern have been investigated as biomarkers to further characterise the tumour phenotype [8].

In our research of novel prognostic markers in TACE candidates we focused on a number of imaging biomarkers reflecting tumour hypoxia-neovascularisation due to their potential impact on the penetration of cytotoxics and post-embolisation ischaemia, with implications in treatment efficacy and patients’ survival. These radiologic parameters include the presence of arterial ectatic neovascularisation (AEN), peri-tumour capsule (PTC), intra-tumour necrosis (ITN) and artero-venous shunting (AVS). In this pilot study we show that that the presence of ITN, AVS and tumour size predict response to TACE. Moreover, we derived a novel prognostic signature that can be utilized in routine clinical practice to optimise the provision of TACE.

## Methods

### Patients

We conducted a retrospective, multi-institutional study of 98 consecutive patients with a diagnosis of HCC, including 64 treated with conventional TACE at Imperial College, London (UK) between 2001 and 2012 and a second subgroup of 34 patients from Novara (Italy), treated between 2004 and 2013 (Table 1). In both centers TACE consisted of intrarterial infusion of doxorubicin emulsified in lipiodol followed by embolisation with gelatin sponge particles.

**Table 1:** Demographic and clinical characteristics of patients with HCC treated with TACE

Baseline characteristic	*n* = 98, (%) or median, (range)
Age, years	71 (33–84)
Gender	
Male	76 (78)
Female	22 (25)
Risk factors for Chronic Liver Disease	
Hepatitis C Virus infection	32 (33)
Hepatitis B Virus Infection	12 (12)
Ethanol Excess	38 (39)
Others	9 (9)
Unknown	8 (8)
Child Turcotte Pugh Class	
A	76 (77)
B	22 (23)
BCLC Stage	
A	10 (10)
B	77 (78)
C	11 (11)
Number of Nodules	
1–2	77 (79)
> 2	21 (21)
Maximum tumour diameter	
≤ 7 cm	71 (72)
> 7 cm	27 (28)
Portal vein invasion (PVI)	
Absent	87 (89)
Present	11 (11)
Albumin, g/L	33 (14–47)
Total bilirubin, umol/L	17 (4–124)
ALT, IU/L	44 (10–348)
AST, IU/L	59 (20–999)
ALP, IU/L	207 (62–680)
AFP, ng/ml	45 (2–130.000)
INR	1.1 (1.0–1.4)
Platelet Count, × 10^9^/L	133 (46–444)
Number of TACE procedures	
1	33 (34)
2	33 (34)
≥ 3	32 (32)
Prior Treatments	
First line TACE	78 (79)
Resection	7 (6)
Transplantation	1 (1)
Radiofrequency ablation	22 (14)
Modified RECIST response following TACE	
Complete Response	15 (15)
Partial Response	28 (29)
Stable Disease	36 (37)
Progressive Disease	7 (7)
Missing	12 (12)
Peri-tumoural capsule (PTC)	
Absent	81 (83)
Present	17 (17)
Ectatic arterial neovascularization (EAN)	
Absent	10 (10)
Present	88 (88)
Artero-venous shunting (AVS)	
Absent	47 (48)
Present	48 (49)
Not assessable	3 (3)
Intra-tumour necrosis (ITN)	
< 50%	89 (90)
> 50%	9 (10)

**Fig. 1:**
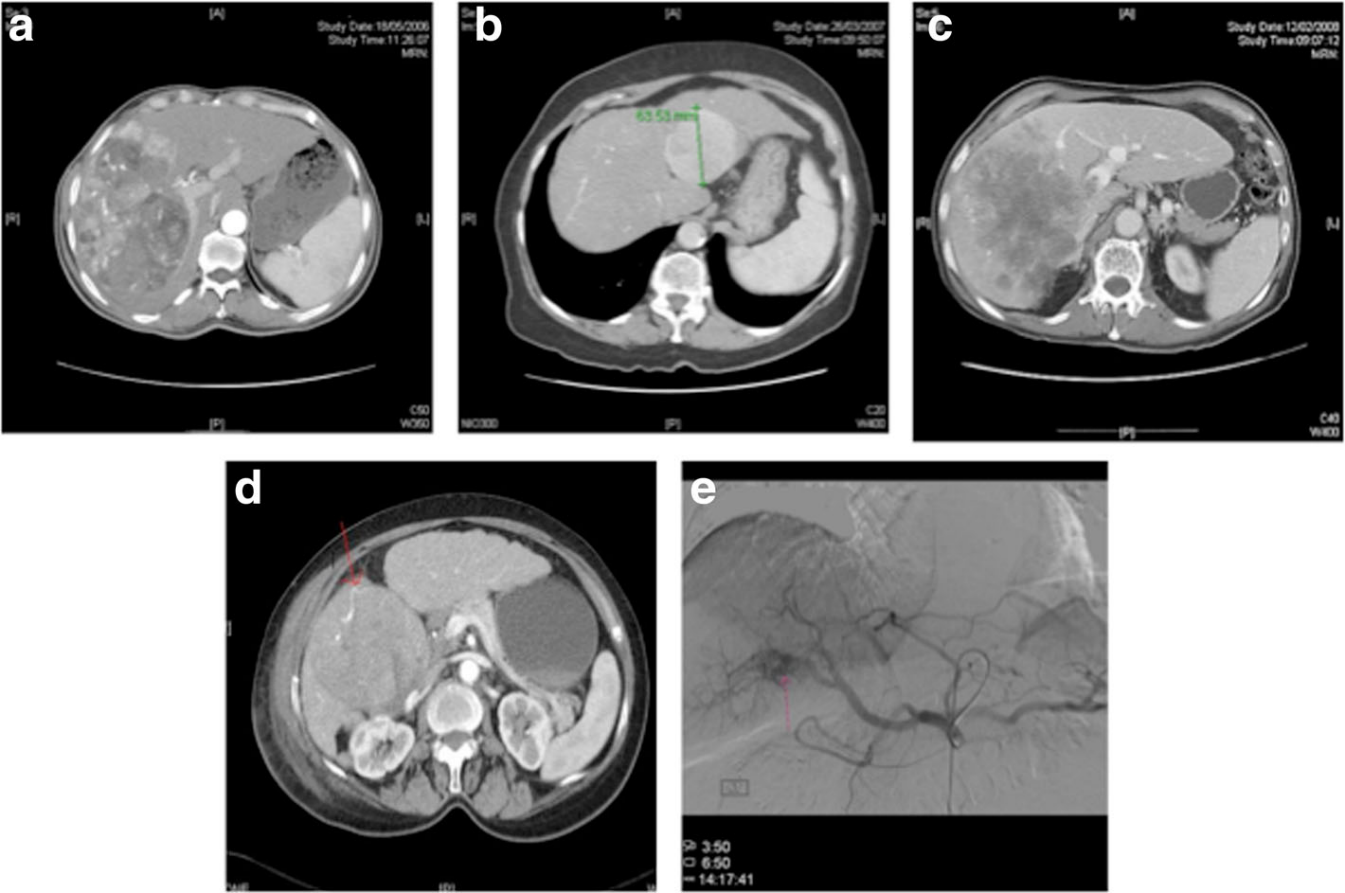
Representative triphasic CT sequences of imaging biomarkers are illustrated: intra-tumour necrosis (ITN) (**a**), presence of peri-tumour capsule (PTC) (**b**), tumour size >7cm with portal vein involvement (PVI) (**c**), arterial ectatic neovascularisation (AEN) (**d**) and artero-venous shunting (AVS) identified on a pre-treatment hepatic arterial angiogram (**e**)

All patients underwent a tri-phasic computer tomography (CT) scan prior to and 6–8 weeks following TACE. A team of hepato-biliary radiologists (P.T. and M.P.) and HPB surgeons (M.P., I.R. and A.G.) blinded to treatment outcome reviewed CT and pre-treatment angiogram images with concordance reached over the qualification of each radiologic feature. Restaging followed modified RECIST (mRECIST) criteria [9]. However, to account for the presence of multiple of multiple lesions being treated within the liver, both targeted and overall imaging responses were assessed. The following radiologic features were evaluated for prognostic significance: size of dominant nodule during arterial enhancement, presence of PTC [10] and ITN if the fraction of tumour lacking arterial enhancement was > 50%. A qualitative analysis of the intra-tumour vascular architecture was performed on arterial CT sequences and matched hepatic arterial angiogram noting the presence of a clear vascular enhancement evidenced by abnormal ectatic vessels running a tortuous course within the tumour mass. The angiographic presence of AVS was defined by arterial to venous contrast extravasation during the arterial phase with a subsequent retained enhancement during the portal and late venous phase. Examples of each radiologic feature are shown (Fig. 1a-f). Assessments of radiologic features were performed on baseline scans. Overall survival (OS) was calculated from initial TACE to the time of death or last-documented follow-up. The local Research Ethics Committee, Imperial College Healthcare NHS Trust, approved the study.

### Statistical analysis

Pearson *χ*^2^-square test and analysis of variance were used to determine any associations between the response to TACE and variables of interest. Kaplan-Meier statistics followed by stepwise backward Cox regression was used for uni- and multivariable analyses of survival. A combined score was derived from the combination of radiologic traits independently associated with patient’s survival based on multivariate Cox regression. All statistical analysis was conducted using SPSS statistical package version 22 (SPSS Inc., Chicago, IL, USA). Variables with a *p*-value greater than 0.10 were removed from the Cox regression model. For all other analyses a significance level of 0.05 was adopted.

## Results

### Demographics

The pre-treatment clinico-pathologic features of the 98 patients identified are reported in Table 1. Most patients were within BCLC-B stage (88%) and Child-Pugh A class (77%). Median age was 64 years (range 33–82), with alcohol excess (39%) and hepatitis C infection (33%) being the most prevalent aetiologies. The majority of patients received TACE as first treatment for HCC (79%), and 66% (*n* = 65) underwent > 1 TACE. In the Hammersmith Hospital cohort 29 from 69 patients (42%) had systemic therapy following TACE. In the majority this consisted of sorafenib (35%) whilst the rest were treated on clinical trial or with chemotherapy. Similarly, 15 patients from the Italian cohort (44%) received sorafenib after TACE. the majority of patients to dominant lesion treated were < 7 cm (72%) with median size of 4.1 cm (range 1–18 cm). When considering the radiologic parameters of interest, nine patients (10%) had tumours displaying > 50% of necrosis, whilst PTC was detected in 17% (*n* = 17). AVS was evident in 49% of patients (*n* = 48), whilst AEN was found in 88% (*n* = 88). Eleven patients (11%) had segmental PVI.

### Radiologic parameters as predictors of treatment response

After the first TACE session, 15 (15%) patients experienced a complete response to therapy, 28 (39%) had partial response, 36 (37%) had stable disease and 7 (7%) had progressive disease according to mRECIST. Response data was not available for 12 patients; one patient died prior to assessment, one underwent transplantation prior to assessment and the remaining 10 were lost to follow-up. In terms of radiological parameters predictive of treatment outcome, a trend was observed between the presence of ITN and poor response to therapy (*p* = 0.08). No other radiologic parameter of interest correlated with treatment response. Tumour size less than 7 cm (*p* = 0.03) and serum alpha-fetoprotein (AFP) < 400 ng/ml (*p* = 0.02) both correlated with improved response with TACE imaging most likely as a reflection of low tumour burden.

### Radiologic parameters as predictors of overall survival

The median follow-up period after TACE was 11 months (2.4–96 months). The OS was 15.4 months (range 2–96 months) with a total of 59 recorded deaths (60%) at the time of censoring. As reported in Table 2, tumour size > 7 cm (*p* < 0.001), presence of AEN (*p* = 0.03), ITN (*p* = 0.02), AFP > 400 ng/ml (*p* = 0.01) and radiologic response (*p* < 0.001) were found to be prognostic on univariate analysis. Neither PTC nor AVS influenced patients’ prognosis. Multivariate analysis identified both the presence of AEN (HR- hazard ratio 4.1 95% CI-confidence interval 1.0–16.5, *p* = 0.04) and radiologic response to initial TACE (HR 0.5 95% CI 0.3–0.8, *p* = 0.01) as significant independent predictors of OS in HCC. A combined prognostic score using both AEN and radiologic response was then derived using logistic regression to determine the predicted probability of death.

**Table 2:** Univariate analysis of prognostic factors of overall survival

UNIVARIATE ANALYSIS				MULTIVARIATE ANALYSIS	
Variable	*N* = 98 (%)	Hazard Ratio (95% CI)	*P*-value	Hazard Ratio (95% CI)	*P*-value
Tumour size					
< 7 cm	23 (36)	2.8 (1.6–5.0)	< 0.001		
> 7 cm	28 (44)				
Number of nodules					
< 2	77 (79)	–	NS		
≥ 2	21 (21)				
AFP, ng/ml					
< 400	83 (85)	2.2 (1.2–4.2)	0.01		
≥ 400	15 (15)				
Peri-tumoural Capsule (PTC)					
Absent	81 (83)	–	NS		
Present	17 (17)				
Ectatic Arterial Neovascularsation (EAN)					
Absent	10 (10)	4.1 (1.0–8.0)	0.03	4.1 (1.0–16.5)	0.04
Present	88 (88)				
Artero-venous shunting (AVS)					
Absent	47 (48)	–	NS		
Present	48 (49)				
Intra-tumour necrosis (ITN)					
< 50%	89 (90)	2.2 (1.1–4.6)	0.02		
≥ 50%	9 (10)				
Portal vein invasion (PVI)					
Absent	10 (10)	–	NS		
Present	88 (88)				
Child Pugh class					
A	76 (77)	–	NS		
B	22 (23)				
BCLC Stage					
A	10 (10)	–	NS		
B	77 (78)				
C	11 (11)				
Radiologic Response					
CR	15 (15)	2.0 (1.4–2.9)	< 0.001	0.5 (0.3–0.8)	0.01
PR	28 (29)				
SD	36 (37)				
PD	7 (7)				

### Assessment of a novel radiologic prognostic signature

Based on the results of the multivariate analysis we derived a compound signature inclusive of tumour size, ITN and AEN, combined with equal weighting (Table 3) and tested this signature for its independent prognostic value in a multivariable Cox regression model including radiologic response and baseline AFP levels. This confirmed mRECIST response (HR 1.9, 95%CI 1.3–2.7, *p* < 0.001) and the radiologic signature (HR 2.0, 95%CI 1.3–2.9, *p* < 0.001) as independent predictors of OS.

**Table 3:** Prognostic factor composing the novel hypoxia-based radiologic signature

Radiologic Feature	Score
Dominant tumour size	
< 7 cm	0
≥ 7 cm	1
Arterial ectatic neovascularization	
Absent	0
Present	1
Intra-tumour necrosis	
Absent	0
Present	1
Good Prognosis: total score 0–1 Poor Prognosis: total score ≥ 2	

**Fig. 2:**
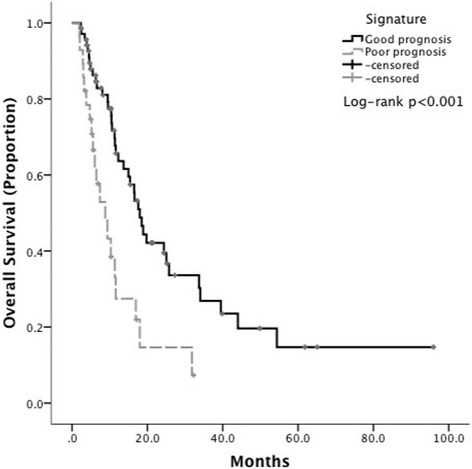
Kaplan-Meier curves showing the relationship between overall survival and the newly qualified radiologic signature in patients treated with TACE

According to baseline prognostic features, nine patients (10%) had 1 adverse factor, whilst 61 (62%) had 2, 21 (21%) had 3 and 7 (7%) had 4. Median OS was not reached in patients with 1 adverse factor, whilst equaled 17.6 months (range 13–21 months) for patients with 2 factors, deteriorating to 9.4 (3.7–15.0) and 7.4 months (5–9.7) for patients with 3 and 4 factors respectively (*p* < 0.001). To facilitate clinical applicability we dichotomised patients as high versus low-risk depending on the presence of ≥ 2 adverse features. Low-risk patients had a median OS of 18 months (range 15–21), deteriorating to 8.8 months (4.7–13) in high-risk patients (HR 2.6, 95%CI 1.4–4.4, p < 0.001). Low-risk patients had a higher proportion of complete and partial responses following TACE (100 and 71%) compared to high-risk (0 and 29%, χ^2^*p* = 0.01) (Fig. 2).

## Discussion

Following decades of improvements in the administration of TACE, research efforts are now concentrated at a more comprehensive clinical phenotyping of patients with intermediate-stage HCC in order to improve patient selection, maximise survival outcomes and prevent iatrogenic morbidity [11].

Our multi-institutional, preliminary study focused upon distinctive radiologic features that are biologically linked to HCC progression through hypoxia and neo-angiogenesis to derive a clinically applicable signature capable of predicting survival advantage after initial TACE.

We demonstrated that patients with tumours > 7 cm, presence of ITN and AEN have a similar outlook to untreated patients with advanced HCC [12], to suggest that TACE-induced survival benefit might have been very small in patients harboring a poor prognostic signature.

Interestingly, patients with good prognosis had a higher proportion of objective radiologic responses to TACE, confirming the ability of the signature to detect a patient subgroup where treatment was more efficacious as a likely result of lower tumour burden and possibly better arterial perfusion of the target lesions.

Importantly, multivariable analysis confirmed the survival advantage identified by the newly qualified signature as independent from other common clinico-pathologic variables including baseline AFP levels and radiologic response.

The prospect of predicting long-term outcomes following TACE based on pre-treatment radiologic features of the tumour is not a novel concept in HCC [13]. However this is the first study to comprehensively evaluate radiologic predictors relating to hypoxia and neo-angiogenesis on routine diagnostic CT scans without the need to extrapolate complex perfusion parameters [14].

Robust evidence suggests that the natural progression of HCC is highly reliant on hypoxia and neo-angiogenesis, both recognised as adverse prognostic domains [15] and therapeutic targets [16]. The reducing oxygen tension that characterizes larger and highly proliferating tumours is the main trigger to hypoxia-inducible factors expression, which leads to the progressive neo-arterialization of nascent HCC nodules through the sustained release of pro-angiogenic factors [17].

Newly formed vasculature demonstrates increased permeability, tortuous course and wider luminal diameters compared to normal vessels, which can be easily detected on arterial CT sequences [14], often in form of aberrant AV shunting [18]. The emergence of ITN is an equally common finding in HCC and a surrogate marker of highly proliferating tumours that fail to maintain the required nutrient and oxygen supply [19]. In our study, AEN and ITN were the only hypoxia-related radiologic traits to display a significant association with patients’ survival, together with tumour size [20].

However, we could not reproduce a prognostic role for segmental PVI or PTC. In a recently published Korean study on 88 patients with prevalently hepatitis B virus related HCC (62%) PVI, major bile ducts invasion and tumour margin irregularity predicted for poorer survival and response to initial TACE [8]. It is documented that survival of patients with small intrahepatic PVI is similar to patients with liver-confined HCC, qualifying them as candidates for TACE [21]. Whilst no information on the extent of PVI is given in the Korean study [8], it is likely that the enriched proportion of patients with limited PVI has contributed to its lack of prognostic significance in our study.

Despite the relatively limited sample size, the multi-center nature of our study supports the significance of our findings, reducing the chances of selection bias stemming from single-institution experience. Central review of diagnostic scans also guarantees for homogeneity and reproducibility in the qualification of imaging biomarkers. Whilst provocative the results of this study require external validation in a large population group with a focus on Eastern patients, where disease aetiology and management differ significantly from Western countries. Another limitation of our study stems from the use of OS, a composite end-point in HCC stemming from the severity of underlying liver disease and cancer progression. OS is also influenced by post-TACE treatment, which however was relatively well balanced across both sub-cohorts. Whilst it could be argued that OS remains the most clinically meaningful endpoint in the management of HCC, the impact of this signature on the progression free survival, a frequently used surrogate of OS in clinical trials, would also be important to expand the translational relevance of our newly qualified prognostic algorithm.

Lastly, a comparative assessment of the radiologic signature with other emerging prognostic models in intermediate-stage HCC would be beneficial to truly appreciate its clinical utility, a task that should be explored in adequately powered, multicenter case series [11].

## Conclusions

To conclude, our study preliminarily qualifies a novel hypoxic-driven signature based on simple and readily accessible radiologic features of HCC including tumour size, AEN and ITN. Given the strong linkage of each biomarker to the biologic foundations of HCC progression, their potential to stratify patients with a 10 months OS difference is not surprising and warrants clinical translation following adequate validation studies. The prognostic relationship with hypoxia and angiogenesis qualifies this signature as a potential stratifying biomarker to optimise therapy, a strategy that should be validated in future studies.

## Abbreviations

AEN: Arterial ectatic neovascularisation
AFP: Alpha-fetoprotein
AVS: Artero-venous shunting
BCLC: “Intermediate” Barcelona Clinic Liver Cancer
CI: Confidence interval
CT: Computer tomography
HCC: Hepatocellular carcinoma
HR: Hazard ratio
ITN: Intra-tumour necrosis
OS: Overall survival
PTC: Peri-tumour capsule
PVI: Portal vein invasion
TACE: Trans-arterial hepatocellular carcinoma
